# Whole-body PET/MRI to detect bone metastases: comparison of the diagnostic performance of the sequences

**DOI:** 10.2478/raon-2024-0062

**Published:** 2024-11-28

**Authors:** Onur Levent Ulusoy, Sadık Server, Murat Yesilova, Nagihan İnan

**Affiliations:** Demiroglu Bilim University, İstanbul, Turkey; Derpartment of Radiology, Florence Nigtingale Hospitals, İstanbul, Turkey

**Keywords:** bone metastases, hybrid imaging, positron-emission tomography, magnetic resonance imaging

## Abstract

**Background:**

Whole-body positron emission tomography/magnetic resonance imaging (WB-PET/MRI) is increasingly used in the initial evaluation of oncology patients. The purpose of this study was to compare the diagnostic performance of WB MRI sequences, attenuation-corrected raw data positron-emission tomography (AC PET), and PET/MRI fused images to detect bone metastases.

**Patients and methods:**

We included 765 consecutive oncologic patients who received WB-PET/MRI from between January 2017 and September 2023. The presence of bone metastases was assessed using the individual sequences by two radiologists. Interobserver agreement was calculated. A receiver operating characteristic (ROC) analysis was performed to assess the performance of each individual sequence and fused images.

**Results:**

Interobserver agreement for the detection of bone metastases on all sequences ranged from good to very good. The reading of the combination of MRI sequences with PET images showed statistically significantly better performance than the reading of individual MRI sequences and PET component only. Contrast enhanced T1 W Volume-interpolated breath-hold examination (CE T1W VIBE) sequence superior to PET for the detection of bone metastasis, but the statistical significance was not as high as with T1W-PET and CE T1W-PET fused images. The highest performance was achieved by the fused CE T1W-PET images with sensitivity of 100%, specificity of 92%, PPV of 96%, and NPV of 100%.

**Conclusions:**

The combination of these CE T1W VIBE sequences with PET images have the highest diagnostic performance in detecting bone metastases in oncologic patients. This sequence should be integrated in WB-PET/MRI acquisitions for initial staging of cancer.

## Introduction

In oncology patients, bone metastases are common, often arising from primary cancers like breast, prostate, lung, and others. Early detection of bone metastasis is critical for accurate staging and optimal treatment. Treatment strategies may involve a combination of systemic therapies, radiation, and supportive care to manage symptoms and improve the quality of life for patients with bone metastases.^1,2,3^

Imaging techniques such as X-rays, bone scans, computed tomography (CT), magnetic resonance imaging (MRI), and positron emission tomography (PET) scans help in detecting and evaluating the extent of bone metastases. X-rays are often used as a first step, while bone scans can reveal areas with increased bone activity. CT and MRI provide detailed images, and PET scans can help detect metastases at an early stage by highlighting abnormal metabolic activity.^4,5,6,7,8^

Whole-body positron emission tomography/magnetic resonance imaging (WB-PET/MRI) is a state of art hybrid imaging technique used in oncology to provide detailed information about both the anatomy and metabolic activity of tissues. It combines the functional information from PET with the detailed structural images from MRI, offering a comprehensive view for oncology patients. The combined data can enhance the accuracy of lesion detection. This integrated approach aids in more accurate diagnosis, staging, and treatment planning for cancer. It may provide better sensitivity and specificity compared to stand-alone modalities. However, the diagnostic performance of PET-MRI sequences for detecting bone metastases can vary depending on factors such as the specific imaging protocol, the type and location of metastases, and the underlying conditions of the patients.^9^

To our knowledge, there is currently no published article comparing the accuracy of WB-PET/MRI sequences in diagnosing bone metastases and work in this area is warranted. The purpose of this study was to compare the diagnostic performance of an individual sequences [pre-contrast T1 weighted (W) Turbo Flash, contrast enhanced T1W Volume-interpolated breath-hold examination (CE T1W VIBE), attenuation-corrected raw data positron-emission tomography (AC PET), and PET/MRI fused images (T1W-PET, CE T1W-PET)] to detect bone metastases in oncology patients.

## Patients and methods

### Patients

Seven hundred sixty-five consecutive patients with histopathologicaly proven primary malignancy who received WB-PET/MRI between January 2017 and September 2023 were evaluated, retrospectively. Two hundred forty-five patients with missing MRI sequences, insufficient image quality, and insufficient data for diagnosis were excluded from the study. As a result, 520 patients with histopathologically proven of their primary malignant tumors by surgery and/or biopsy were included in this research (317 males and 203 females; mean age of 59.27 ± 13.53 years, range 21–83 years). Among these patients, 76 (14.62%) of them had bone metastases (53 males and 23 females; mean age of 56.57±14.60 years, range 24–72); 444 of them had no bone metastases. A total of 152 bone metastases in 76 patients were included in the final evaluation.

The study was approved by the ethics committee (The ethical approval number: 2024/177) and because it was a retrospective study, written permission was not required.

### Imaging protocol

After fasting for at least 6 hours, the blood glucose level was assessed with a blood glucose meter (One Touch Vita; Life Scan, Milpitas, CA, USA) before imaging to ensure that it was <140 mg/dl. WB-PET/MRI was performed 45 ± 10 minutes after ^18^F-Fluorodeoxyglucose (FDG) injection (average dosages, 4.541 MBq/kg weight; spectrum, 370–400 MBq). The WB-PET/MRI images were acquired in supine position on a 3 tesla Biograph mMR scanner (Siemens Healthcare, Erlangen, Germany) using a 16-channel head and neck surface coil, three 12-channel body coils, and 56 lutetium oxy orthosilicate avalanche photodiode PET detector blocks. These body coils were combined to form a multichannel WB coil by using the total imaging matrix technology. The WB images were obtained in 5 to 6 bed positions according to the size of the patient and each bedtime position was maintained between 2 and 2.5 min. In all patients, the WB PET/MRI covered the entire body from head to knee. For the attenuation correction, 4-point Dixon images were obtained in the coronal plane. The comprehensive MRI protocol consisted of T2-W single-shot echo train (HASTE; TR/TE, 1,500 ms/87 ms) and T1-W slice-selective Turbo Flash (TR/TE, 1,600 ms/2.5 ms) in the axial planes. PET acquisition occurred simultaneously during the WB MRI acquisition. Following the precontrast images, a gadolinium-based contrast agent [Dotarem^®^(Gadoterate Meglumine)] was used to obtain breath-hold 3D VIBE dynamic postcontrast images (TR/TE, 4.56 ms/2.03 ms) covering the upper abdomen in the arterial, portal venous, and late venous phases. Following the acquisition of the dynamic upper abdominal images, continuous breath-hold 3D VIBE images were acquired in the axial plane from head to knee. All the sections were combined, resulting in uninterrupted WB coverage. The total scan duration of the WB-PET/MRI examination was 50–60 min.

### Imaging evaluation

In our research, two radiologists (S.S., and N.I.), one had 15 years of experience, and the other had 20 years of experience in reading MRI and both had 8 years of experience in reading hybrid imaging, performed all readings, in consensus. The presence of bone involvement on the individual sequences [pre-contrast T1W, CE T1W VIBE, AC PET, and PET/MRI fused images (T1W-PET, CE T1W-PET)] were reviewed separately, in a random order and at 1-month intervals to avoid any recall bias. The following widely accepted findings were applied to determine the presence of bone metastasis. Normal marrow was defined on T1W images as the homogeneous signal intensity that was higher than that of discs and muscles. A focal bone metastasis was defined by low signal intensity on T1W (lower than or equal to the signal intensity of discs or muscles), showing contrast enhancement, and pathologic FDG activation on PET images.^10^ Discrepant findings were resolved by consensus decision making in a separate session between the readers. The readers were blinded to patient identity, status, and clinical and biological data.

The reference standard for bone metastases was constructed in consensus by the readers along with a third reader who had 22 years’ experience in reading musculoskeletal MRI (O.L.U.). The third reader reviewed all baseline and follow-up CT or MRI examinations (6.2±1.6 month, and histopathological data). Increase or decrease in size of lesions after therapy or newly occurred cortical destruction were regarded as signs of malignancy.

False-positive and false-negative findings of any reading were assessed during the consensus reading by two readers. False-positive findings were degenerative disease, vertebral hemangioma, fracture, focal bone marrow hyperplasia, and diffuse heterogeneous or hyperplastic bone marrow; false-negative findings were sclerotic lesions, poor contrast between lesions and surrounding hypercellular bone marrow.^11^

**FIGURE 1.**
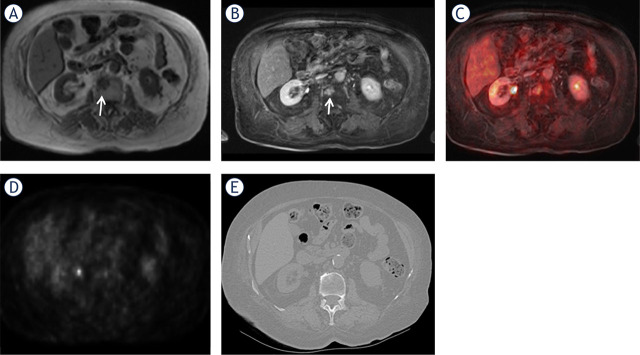
A 53-year-old woman with breast cancer. A metastasis can be observed in the L2 vertebral body (arrows) on the precontrast T1 weighted (W) image **(A)**, contrast enhanced (CE) T1W image **(B)**, and CE T1W VIBE-PET fused image **(C)**. The AC PET axial image **(D)** does not show FDG uptake. CT yielded false-negative results **(E)**. AC PET = attenuation-corrected raw data positron-emission tomography; FDG = fluorodeoxyglucose; VIBE = volume-interpolated breath-hold examination

### Statistical analysis

Statistical analyses were performed using Statistical Package for Social Sciences for Windows software version 25 (IBM Corp.; Armonk, NY, USA). Interobserver agreement for each MR sequence between the two readers was assessed. The degree of agreement was determined by using the kappa value, and categorized as follows: 0–0.20, slight agreement; 0.21–0.40, fair agreement; 0.41–0.60, moderate agreement; 0.61–0.80, good agreement; and 0.81–1.00, excellent agreement.

The variables were investigated using the Kolmogorov-Smirnov test to determine whether the distribution was normal. Because most variables except for age were not normally distributed, Fried man’s test was conducted to evaluate whether there was a significant change in the total number of detected bone metastasis among the different sequences.

A receiver operating characteristic (ROC) analysis was performed to assess the performance of each individual sequence and fused images according to the reference standard. The area under the curve (AUC) was reported along with a 95% confidence interval (CI). The sensitivity, specificity, positive predictive value (PPV), and negative predictive value (NPV) were calculated. Finally, pairwise comparisons of the AUC values were performed to rank the individual sequences and fused images according to diagnostic accuracy, using a chi-squared test of equality of ROC curves’ areas. A p value < 0.05 indicates statistical significance for all tests.

## Results

### Patient characteristics

Table 1 shows tumor histopathologic features. Primary malignancies included lung cancer (*n* =26), hepatobiliary carcinoma (*n* =12), genitourinary carcinoma (*n* = 8), gastrointestinal cancer (*n* = 7), and breast cancer (*n* = 23). Metastases were in the ribs (*n* = 18), sternum (*n* =7), pelvic bones (*n* = 23), femur (*n* =13), cervical (*n* = 19), thoracic (*n* =22), lumbar (*n* = 32), and sacral vertebrae (*n* = 18).

**TABLE 1.** Tumor histopathologic features

**Primary malignant tumors**	**n**
Hepatobiliary	12
Gastrointestinal	7
Genitourinary	8
Breast	23
Lung	26

### Inter-observer agreement

Interobserver agreement for the detection of bone metastases on all sequences was ranged from good to very good (Table 2).

**TABLE 2.** Inter-observer variability for the detection of bone metastases on all sequences

**Sequences**	**kappa**	**95 %CI**
**Precontrast T1W**	0.86	0.69–0.92
**CE T1W VIBE**	0.87	0.77–0.94
**AC PET**	0.83	0.76–0.90
**T1W-PET**	0.86	0.78–0.91
**CE T1W-PET**	0.88	0.82–0.94

AC PET = attenuation-corrected raw data positron-emission tomography; CE = contrast enhanced; VIBE = volume-interpolated breath-hold examination

### Diagnostic performance of sequences

The results on diagnostic performance of sequences, PET images, and fused images are summarized in Table 3. The reading of the combination of MRI sequences with PET images showed statistically significantly better performance than the reading of individual MRI sequences and PET component only. CE T1W MRI superior to PET for the detection of bone metastasis, but the statistical significance was not as high as with T1W-PET and CE T1W-PET. The highest performance was achieved by the fused CE T1W MRI/PET images with sensitivity of 100%, specificity of 92%, PPV of 96%, and NPV of 100%.

**TABLE 3.** Diagnostic performance of sequences

**Sequences**	**Sensitivity (%)**	**Specificity (%)**	**95%CI**	**AUC**	**P**	**PPV (%)**	**NPV (%)**
**Precontrast T1W**	78	50	63.2–78.3	0.587 ± 0.064	0.191	75	54
**CE T1W VIBE**	82	58	67.3–88.9	0.751 ± 0.065	0.024	79	63
**AC PET**	80	54	67.9–79.4	0.667 ± 0.065	0.013	78	58
**T1W-PET**	84	61.5	72.2–91.9	0.796 ± 0.063	0.003	81	67
**CE T1W-PET**	100	92	74.0–99.8	0.952 ± 0.067	0.001	96	100

AC PET = attenuation-corrected raw data positron-emission tomography; AUC = area under the curve; CE = contrast enhanced; CI = confidence interval; NPV = negative predictive value; PPV = positive predictive value; VIBE = volume-interpolated breath-hold examination

## Discussion

Among the various imaging modalities currently available to detect bone metastasis, hybrid techniques with ^18^F-FDG PET/CT or PET/MRI which fuse morphological and functional data are the most sensitive and specific. In these hybrid techniques, PET/CT is used much more widely due to its short imaging time advantage and easy accessibility. For this reason, there are many studies comparing the diagnostic sensitivity of PET/CT with other methods. As a result, the superiority of PET/CT for the detection of metastases was reported in most of these studies.^12,13,14,15,16^ A meta-analysis including 145 studies compared ^18^F-FDG PET/CT, CT, MRI, and bone scintigraphy for the detection of bone metastases.^17^ The results indicated sensitivity and specificity of PET and MRI higher than for CT and bone scintigraphy alone. While ^18^F-FDG PET/CT was reported higher sensitivity for osteolytic metastases, the same is not true for osteoblastic metastases. The reason for this might be the different uptake mechanism in osteolytic and osteoblastic bone metastases. Osteoblast activity resulting increase of bone matrix and decrease in cell density resulting lower FDG activation.^18,19^ Hence, the diagnostic value of PET/CT will decrease and that of MRI will increase, especially in these osteoblastic metastases.

There are some studies comparing the performance of MRI sequences with PET/CT images.^20,21,22,23^ For examples, Jambor *et al*. compared the diagnostic accuracy of ^99m^Tc-hydroxymethane diphosphonate (^99m^Tc-HDP) planar bone scintigraphy, ^99m^Tc-HDP SPECT, ^99m^Tc-HDP SPECT/CT, ^18^F-NaF PET/CT and whole-body MRI, including diffusion weighted imaging, DWI for the detection of bone metastases in high risk breast and prostate cancer patients.^21^ As a result, the authors shown that WB MRI DWI and ^18^F-NaF PET/CT being superior to conventional nuclear imaging techniques for discovering bone metastases. They were also found that, WB MRI DWI was as accurate as ^18^F-NaF PET/CT for the detection of bone metastases. Considering the cost, availability and radiation dose, WB MRI DWI may be a preferred choice in comparison with ^18^F-NaF PET/CT. In this study, it was also reported that the sensitivity of PET CT was lower, especially in hypometabolic osteoblastic metastases. Therefore, WB-PET/MRI may be more sensitive in detecting metastases with hypometabolic activity, due to the superior soft tissue resolution of MRI.

There are few studies investigating the diagnostic value of WB-PET/MRI in detecting bone metastases.^24,25,26,27,28,29,30,31,32^ A study has shown that the overall performance of PET/MRI and PET/CT was equivalent for detection and characterization of bone lesions. However, lesion delineation of PET-positive findings was superior in PET/MR imaging with diagnostic T1W TSE or T1W Dixon in-phase sequences compared with PET/CT. This finding might be clinically important for bone metastases with low uptake on PET.^24^ Beiderwellen and colleagues examined total of 75 bone lesions, of which 48 lesions were metastases, and 27 lesions were benign.^25^ The results indicated that PET/MRI allowed identification of all bone metastases, while PET/CT identified 45 of 48 bone metastases correctly (94%). In benign lesions, PET/CT outperformed PET/MRI by correctly identifying 96% bone lesions compared with 67% in PET/MRI. The benign lesions were missed by PET/MRI consisted of PET negative osteosclerotic lesions. A retrospective study comparing 68 Ga-PSMA PET/MRI and PET/CT in prostate cancer showed higher conspicuity of bone lesions on MRI compared with CT (p < 0.006). In conclusion, it was reported PET/MRI has excellent diagnostic performance in evaluating osseous metastases.^32^

As a result of these few studies, the diagnostic superiority of PET/MRI has been reported. However, there is no study comparing the performance of PET/MRI sequences for bone metastases. Our data showed that the CE T1W-PET fused images showed superior lesion detection rate than only PET component. This may reflect the superiority of PET/MRI over PET/CT with its ability to assess early infiltration of bone marrow with malignant tissue with the excellent soft tissue contrast of MRI. Tumor proliferation in the bone marrow results in hypointense T1 and hyperintense T2 signal, as well as relatively strong contrast media uptake, regularly seen in osteolytic disease. In contrast to these findings, the described signal changes might be less pronounced or even absent in osteoblastic metastases because of the lower tumor cellularity.^10,33^ The use of morphologic and functional MR imaging techniques enables the assessment of complementary data in bone metastases and increases the accurate assignment of PET-positive findings to anatomic structures.

Our study has limitations, including the limited number of patients and lack of histopathologic confirmation for every lesion. The results should be considered as preliminary and larger studies are needed to show the potential of FDG-PET/MR. As we know, DWI has high diagnostic accuracy for detecting bone metastases. However, we routinely obtain WB T1W, T2W, CE T1W images, dynamic liver and postcontrast 3D T1W brain images for evaluating of oncologic patients. DWI sequences are not routinely taken in order not to prolong the imaging time further. Therefore, we could not analyze the diagnostic accuracy of DWI images. This is one of the limitations of our study.

For detecting bone metastases, the differences between MRI-based attenuation correction (AC) in PET/MRI and CT-derived AC in PET/CT are especially pronounced. Bone metastases detection relies on accurate AC, and the artifacts in MRI-based AC can significantly impact accuracy and reliability. MRI primarily captures soft tissues based on proton density, and bone, especially cortical bone, produces very low MRI signal. This results in misclassification of bone as soft tissue or air, leading to underestimation of attenuation in bone-dense regions. CT directly measures tissue densities, including bone, providing accurate attenuation values for both cortical and trabecular bone. This makes CT-derived AC highly reliable for bone metastases detection, as bone attenuation is appropriately accounted for. Lesions in bone-rich areas are more likely to be detected and quantified accurately because the higher attenuation of bone is correctly incorporated into the PET images. PET/MRI is more prone to missing metastases near bone-air interfaces (e.g., skull), whereas PET/CT provides better accuracy in these regions. MRI-based AC is more affected by metal artifacts, reducing lesion detectability near metal implants, while CT-based AC is more robust.^34,35^

Replacing PET/MRI with a combination of sequential MRI and PET/CT for lesion detection is a topic of interest in clinical imaging, as each modality offers unique advantages. PET/MRI has the advantage of providing both metabolic and anatomical data in a single session, which can streamline the patient experience and reduce total scan time. This simultaneous acquisition can be especially useful in detecting lesions in soft tissues, such as in neuroimaging (e.g., brain tumors), liver, or prostate, where soft-tissue contrast is critical. Conducting two separate imaging sessions (MRI and PET/CT) requires more logistical coordination and may be time-consuming for the patient. In clinical practice, many institutions already perform PET/CT followed by targeted MRI for specific regions (e.g., brain, liver, prostate), so this workflow is already well established. While MRI provides the same soft-tissue contrast as in PET/MRI, the lack of simultaneous acquisition can sometimes lead to misalignment between PET and MRI data, particularly in organs prone to motion (e.g., lungs, abdomen). But CT-based AC offers better accuracy for bone and air interfaces, leading to more accurate PET quantification, especially in whole-body oncological imaging and detection of bone metastases. PET/MRI offers a lower radiation dose compared to sequential MRI and PET/CT, making it more attractive for cases where minimizing radiation exposure is crucial.^34,35^

In addition to commonly used radionuclides like ^99m^Tc and ^18^F-FDG, Fluorine-18 Sodium Fluoride (^18^F-NaF) is highly sensitive for detecting bone metastases because it is rapidly incorporated into the bone matrix at sites of osteoblastic activity, which is typically elevated in metastatic bone lesions. ^18^F-NaF PET/CT provides higher resolution and more precise localization of bone metastases compared to traditional bone scintigraphy using ^99m^Tc. It is particularly advantageous in patients where early detection is crucial, such as those with breast, prostate, and lung cancers that commonly metastasize to the bone.^36^

## Conclusions

In conclusion, our results showed that FDG-PET/MRI may be beneficial in patients with primary malignancy to detect early bone metastasis without radiation exposure. The metabolic information from PET data together with the diagnostic accuracy of CE T1W-PET fused images may increase the sensitivity of detection.

## References

[1] Yu H, Tsai YY, Hoffe SE (2012). Overview of diagnosis and management of metastatic disease to bone. Cancer Control.

[2] Baüerle T, Semmler W (2009). Imaging response to systemic therapy for bone metastases. Eur Radiol.

[3] Vassiliou V, Andreopoulos D, Frangos S, Tselis N, Giannopoulou E, Lutz S (2011). Bone metastases: assessment of therapeutic response through radiological and nuclear medicine imaging modalities. Clin Oncol.

[4] O’Sullivan GJ, Carty FL, Cronin CG (2015). Imaging of bone metastasis: an update. World J Radiol.

[5] Even-Sapir E, Metser U, Mishani E (2006). The detection of bone metastases in patients with high-risk prostate cancer: ^99m^Tc-MDP planar bone scintigraphy, single- and multi-field-of-view SPECT, ^18^F-fluoride PET, and ^18^F-fluoride PET/CT. J Nucl Med.

[6] Römer W, Nömayr A, Uder M, Bautz Werner, Kuwert Torsten (2006). SPECT-guided CT for evaluating foci of increased bone metabolism classified as indeterminate on SPECT in cancer patients. J Nucl Med.

[7] Utsunomiya D, Shiraishi S, Imuta M, Tomiguchi S, Kawanaka K, Morishita S (2006). Added value of SPECT/CT fusion in assessing suspected bone metastasis: comparison with scintigraphy alone and nonfused scintigraphy and CT. Radiology.

[8] Schmidt GP, Schoenberg SO, Schmid R, Stahl R, Tiling R, Becker CR (2007). Screening for bone metastases: whole-body MRI using a 32-channel system versus dual-modality PET-CT. Eur Radiol.

[9] Kogan F, Broski SM, Yoon D, Gold GE (2018). Applications of PET-MRI in musculoskeletal disease. J Magn Reson Imaging.

[10] Vanel D, Dromain C, Tardivon A (2000). MRI of bone marrow disorders. Eur Radiol.

[11] Padhani AR, Koh DM, Collins DJ (2011). Whole-body diffusion weighted MR imaging in cancer: current status and research directions. Radiology.

[12] Hildebrandt MG, Gerke O, Baun C, Falch K, Hansen JA, Farahani ZA (2016). [181F] fluorodeoxyglucose (FDG)-positron emission tomography (PET)/computed tomography (CT) in suspected recurrent breast cancer: a prospective comparative study of dual-time-point FDG-PET/CT, contrast-enhanced CT, and bone scintigraphy. J Clin Oncol.

[13] Park S, Yoon J-K, Lee SJ, Kang SY, Yim H, An Y-S (2017). Prognostic utility of FDG PET/CT and bone scintigraphy in breast cancer patients with bone-only metastasis. Medicine.

[14] Du Y, Cullum I, Illidge TM, Ell PJ (2007). Fusion of metabolic function and morphology: sequential [^18^F] fluorodeoxyglucose positron-emission tomography/computed tomography studies yield new insights into the natural history of bone metastases in breast cancer. J Clin Oncol.

[15] Krüger S, Buck AK, Mottaghy FM, Hasenkamp E, Pauls S, Schumann C (2009). Detection of bone metastases in patients with lung cancer: ^99m^Tc-MDP planar bone scintigraphy, ^18^F-fluoride PET or ^18^F-FDG PET/CT. Eur J Nucl Med Mol Imaging.

[16] Hahn S, Heusner T, Kümmel S, Köninger A, Nagarajah J, Müller S (2011). Comparison of FDG-PET/CT and bone scintigraphy for detection of bone metastases in breast cancer. Acta Radiol.

[17] Yang HL, Liu T, Wang XM, Xu Y, Deng SM (2011). Diagnosis of bone metastases: a meta-analysis comparing ^18^FDG PET, CT, MRI and bone scintigraphy. Eur Radiol.

[18] Huyge V, Garcia C, Vanderstappen A, Alexiou J, Gil T, Flamen P (2009). Progressive osteoblastic bone metastases in breast cancer negative on FDG-PET. Clin Nucl Med.

[19] Nakai T, Okuyama C, Kubota T, Yamada K, Ushijima Y, Taniike K (2005). Pitfalls of FDG-PET for the diagnosis of osteoblastic bone metastases in patients with breast cancer. Eur J Nucl Med Mol Imaging.

[20] Liu T, Cheng T, Xu W, Yan WL, Liu J, Yang HL (2011). A metanalysis of ^18^FDG-PET, MRI and bone scintigraphy for diagnosis of bone metastases in patients with breast cancer. Skeletal Radiol.

[21] Jambor I, Kuisma A, Ramadan S, Huovinen R, Sandell M, Kajander S (2016). Prospective evaluation of planar bone scintigraphy, SPECT, SPECT/CT, ^18^F-NaF PET/CT and whole body 1.5T MRI, including DWI, for the detection of bone metastases in high risk breast and prostate cancer patients: SKELETA clinical trial. Acta Oncol.

[22] Ghanem N, Uhl M, Brink I, Schäfer O, Kelly T, Moser E (2005). Diagnostic value of MRI in comparison to scintigraphy, PET, MS-CT and PET/CT for the detection of metastases of bone. Eur J Radiol.

[23] Qu X, Huang X, Yan W, Wu L, Dai K (2012). A meta-analysis of ^18^FDG-PET–CT, ^18^FDG-PET, MRI and bone scintigraphy for diagnosis of bone metastases in patients with lung cancer. Eur J Radiol.

[24] Eiber M, Takei T, Souvatzoglou M, Fürst S, Gaertner FC, Loeffelbein DJ (2014). Performance of whole-body integrated ^18^F-FDG PET/MR in comparison to PET/CT for evaluation of malignant bone lesions. J Nucl Med.

[25] Beiderwellen K, Huebner M, Heusch P, Grueneisen J, Ruhlmann V, Nensa F (2014). Whole-body [(1)(8)F]FDG PET/MRI vs. PET/CT in the assessment of bone lesions in oncological patients: initial results. Eur Radiol.

[26] Löfgren J, Mortensen J, Rasmussen SH, Madsen C, Loft A, Hansen AE (2017). A prospective study comparing ^99m^Tc-hydroxyethylene-diphosphonate planar bone scintigraphy and whole-body SPECT/CT with ^18^F-fluoride PET/CT and ^18^F-fluoride PET/MRI for diagnosing bone metastases. J Nucl Med.

[27] Bruckmann NM, Kirchner IJ, Umutlu L, Fendler WF, Seifert R, Hermann K (2021). Prospective comparison of the diagnostic accuracy of ^18^F-FDG PET/MRI, MRI, CT, and bone scintigraphy for the detection of bone metastases in the initial staging of primary breast cancer patients. Eur Radiol.

[28] Catalano OA, Nicolai E, Rosen BR, Luongo A, Catalano M, Iannace C (2015). Comparison of CE FDG-PET/CT with CE-FDG-PET/MR in the evaluation of osseous metastases in breast cancer patients. Br J Cancer.

[29] Sonni I, Minamimoto R, Baratto L, Gambhir SS, Loening AM, Vasanawala SS (2020). Simultaneous PET/MRI in the evaluation of breast and prostate cancer using combined Na[^18^F] F and [^18^F]FDG: a focus on skeletal lesions. Mol Imaging Biol.

[30] Sawicki LM, Grueneisen J, Schaarschmidt BM, Buchbender C, Nagarajah J, Umutlu L (2016). Evaluation of ^18^F-FDG PET/MRI, ^18^F-FDG PET/CT, MRI, and CT in whole-body staging of recurrent breast cancer. Eur J Radiol.

[31] Samarin A, Hullner M, Queiroz MA, Stolzmann P, Burger IA, Schulthess G (2015). ^18^F-FDG-PET/MR increases diagnostic confidence in detection of bone metastases compared with ^18^F-FDG-PET/CT. Nucl Med Commun.

[32] Freitag MT, Radtke JP, Hadaschik BA, Kopp-Schneider A, Eder M, Kopka K (2016). Comparison of hybrid ^68^Ga-PSMA PET/MRI and ^68^Ga-PSMA PET/CT in the evaluation of lymph node and bone metastases of prostate cancer. Eur J Nucl Med Mol Imaging.

[33] Steinborn MM, Heuck AF, Tiling R, Bruegel M, Gauger L, Reiser M (1999). Whole-body bone marrow MRI in patients with metastatic disease to the skeletal system. J Comput Assist Tomogr.

[34] Zhan Y, Zhang G, Li M, Zhou X (2021). Whole-body MRI vs. PET/CT for the detection of bone metastases in patients with prostate cancer: a systematic review and meta-analysis. Front Oncol.

[35] Bashir U, Mallia A, Stirling J, Joemon J, MacKewn J, Charles-Edwards G (2015). PET/MRI in oncological imaging: state of the art. Diagnostics.

[36] Araz M, Aras G, Kucuk ON (2015). The role of ^18^F-NaF PET/CT in metastatic bone disease. J Bone Oncol.

